# High-throughput single-cell DNA sequencing of acute myeloid leukemia tumors with droplet microfluidics

**DOI:** 10.1101/gr.232272.117

**Published:** 2018-09

**Authors:** Maurizio Pellegrino, Adam Sciambi, Sebastian Treusch, Robert Durruthy-Durruthy, Kaustubh Gokhale, Jose Jacob, Tina X. Chen, Jennifer A. Geis, William Oldham, Jairo Matthews, Hagop Kantarjian, P. Andrew Futreal, Keyur Patel, Keith W. Jones, Koichi Takahashi, Dennis J. Eastburn

**Affiliations:** 1 Mission Bio, Incorporated, South San Francisco, California 94080, USA;; 2 Department of Leukemia, The University of Texas MD Anderson Cancer Center, Houston, Texas 77030, USA;; 3 Department of Genomic Medicine, The University of Texas MD Anderson Cancer Center, Houston, Texas 77030, USA;; 4 Department of Hematopathology, The University of Texas MD Anderson Cancer Center, Houston, Texas 77030, USA

## Abstract

To enable the characterization of genetic heterogeneity in tumor cell populations, we developed a novel microfluidic approach that barcodes amplified genomic DNA from thousands of individual cancer cells confined to droplets. The barcodes are then used to reassemble the genetic profiles of cells from next-generation sequencing data. By using this approach, we sequenced longitudinally collected acute myeloid leukemia (AML) tumor populations from two patients and genotyped up to 62 disease relevant loci across more than 16,000 individual cells. Targeted single-cell sequencing was able to sensitively identify cells harboring pathogenic mutations during complete remission and uncovered complex clonal evolution within AML tumors that was not observable with bulk sequencing. We anticipate that this approach will make feasible the routine analysis of AML heterogeneity, leading to improved stratification and therapy selection for the disease.

Current tumor sequencing paradigms are inadequate to fully characterize many instances of acute myeloid leukemia (AML) (Griffith et al. 2015; Paguirigan et al. 2015). A major challenge has been the unambiguous identification of potentially rare and genetically heterogeneous neoplastic cell populations, with subclones capable of critically impacting tumor evolution and the acquisition of therapeutic resistance (Welch et al. 2012; Saito et al. 2017; Smith et al. 2017). Standard bulk population sequencing is often unable to identify rare alleles or definitively determine whether mutations co-occur within the same cell. Single-cell sequencing has the potential to address these key issues and transform our ability to accurately characterize clonal heterogeneity in AML; however, previous single-cell studies examining genetic variation in AML have relied upon laborious, expensive, and low-throughput technologies that are not readily scalable for routine analysis of the disease.

An established approach for high-throughput and scalable single-cell sequencing uses cell-identifying barcodes to tag the nucleic acids of individual cells confined to emulsion droplets (Klein et al. 2015; Macosko et al. 2015; Rotem et al. 2015; Lan et al. 2017). Although it is now feasible to perform single-cell RNA-seq on thousands of cells using this type of approach, high-throughput single-cell DNA genotyping using droplet microfluidics has not been demonstrated on eukaryotic cells. This is primarily due to the challenges associated with efficiently lysing cells, freeing genomic DNA from chromatin and enabling efficient amplification in the presence of high concentrations of crude lysate (Jackson et al. 1990; Strauss 2001).

In this report, we present a microfluidic approach, relying on cell-identifying molecular barcodes, that overcomes existing barriers to high-throughput single-cell DNA sequencing. We focus our single-cell sequencing analysis on 62 genomic loci implicated in the acquisition or progression of AML. As a demonstration of the technology, we characterize longitudinal samples from AML patients and uncover features of clonal architecture that are not available from bulk sequencing data. This rapid, cost-effective, and scalable approach promises to make routine analysis of genetic variation in tumors a reality.

## Results

### Droplet workflow for genomic DNA amplification and barcoding

**Figure 1.**
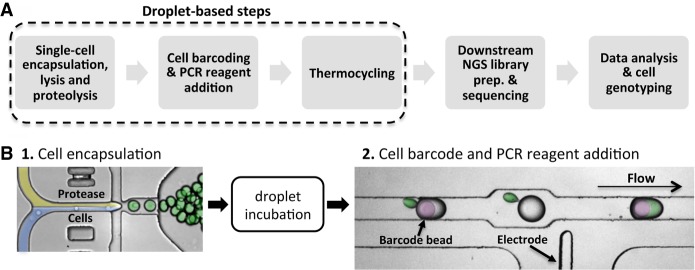
Protease-based droplet workflow for single-cell genomic DNA amplification and barcoding. (*A*) Overview of the steps in the workflow. (*B*) Microfluidic devices to perform the two-step droplet workflow. Cells (pseudocolored in blue) are first encapsulated with lysis buffer containing protease (yellow) and incubated to promote proteolysis (green droplets). Protease activity is then thermally inactivated, and the droplets containing the cell lysate are paired and merged with droplets containing PCR reagents and molecular barcode-carrying hydrogel beads (pseudocolored in purple).

**Figure 2.**
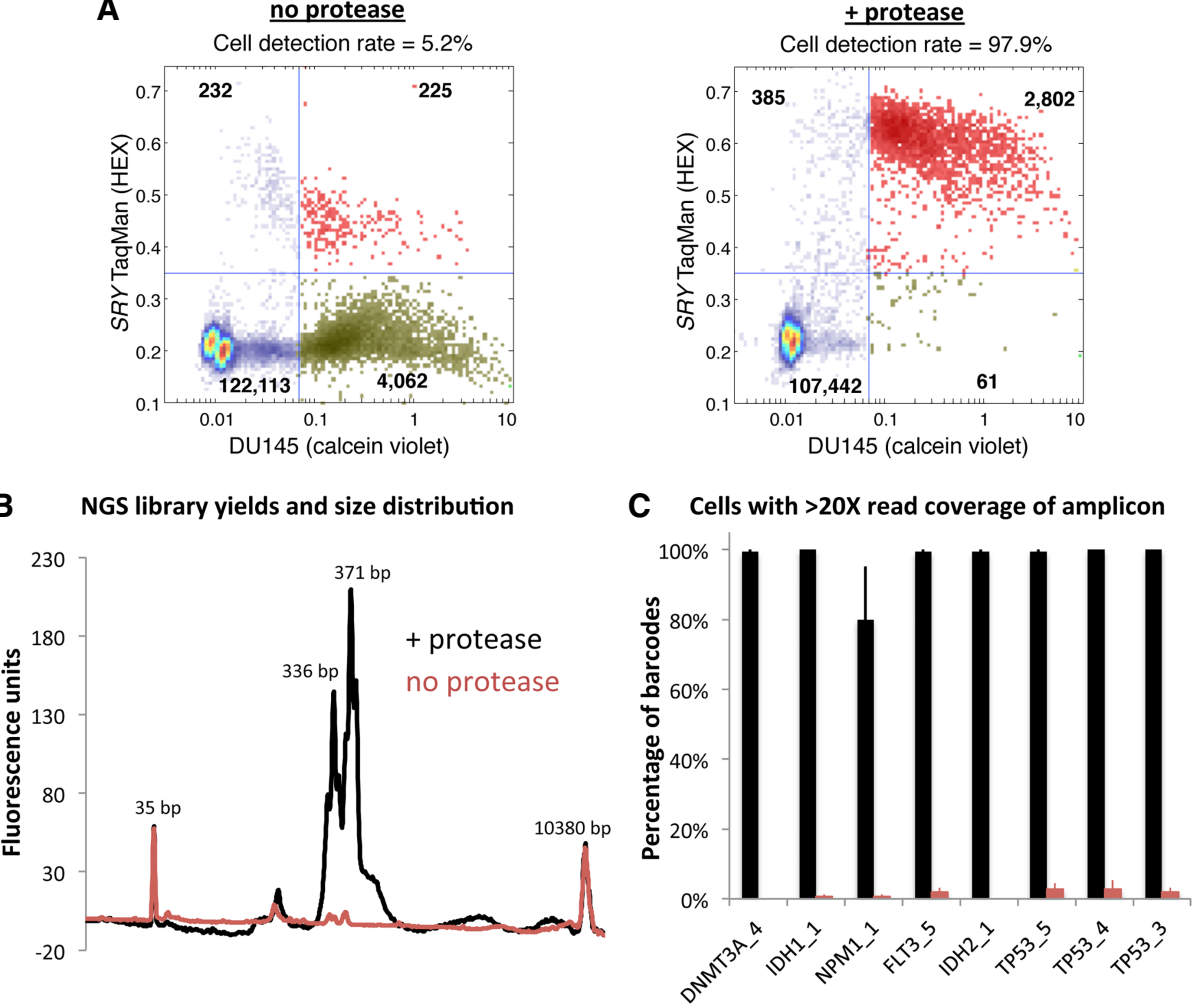
Protease-based workflows provide improved genomic DNA amplification. (*A*) When protease enzyme is left out of the workflow for single-cell gDNA PCR in droplets, only ∼5% of DU145 cells (viability stained on the *x*-axis) are positive for *SRY* TaqMan reaction fluorescence (*y*-axis). Using protease during cell lysis improves the DU145 cell detection rate to ∼98% (red points in *upper right* quadrant). Points in the plot represent droplets. (*B*) Bioanalyzer traces of sequencing libraries prepared from cells processed through the workflow with (black trace) or without (red trace) the use of protease indicate that PCR amplification in droplets is improved with proteolysis. The two-step workflow with protease enables better sequencing coverage depth per cell across the eight amplified target loci listed on the *x*-axis (*C*).

To enable the characterization of genetic diversity within cancer cell populations, we developed a novel two-step microfluidic droplet workflow that enables efficient and massively parallel single-cell PCR-based barcoding (Fig. 1A,B). The microfluidic workflow first encapsulates individual cells in droplets, lyses the cells, and prepares the lysate for genomic DNA amplification using proteases. Following this lysate preparation step, the proteases are inactivated via heat denaturation, and droplets containing the genomes of individual cells are paired with cell-identifying barcodes and PCR amplification reagents. To demonstrate the advantage of the protease in the two-step workflow, we performed droplet-based single-cell TaqMan PCR reactions targeting the *SRY* locus on the Y Chromosome, present as a single copy in a karyotypically normal cell (Fig. 2A). We carried out PACS (PCR-activated cell sorting) on calcein violet–stained DU145 prostate cancer cells encapsulated and lysed with or without the addition of a protease (Eastburn et al. 2014; Pellegrino et al. 2016). In the absence of protease during cell lysis, only 5.2% of detected DU145 cells were positive for TaqMan fluorescence. The inclusion of the protease resulted in a dramatically improved *SRY* locus detection rate of 97.9%.

We next sought to determine if the two-step workflow was also required for single-cell barcoding of amplicons targeting eight genomic loci located in *TP53*, *DNMT3A*, *IDH1*, *IDH2*, *FLT3*, and *NPM1*. To do this, we synthesized hydrogel beads with oligonucleotides containing both cell-identifying barcodes and different gene specific primer sequences (Klein et al. 2015). These barcoded beads were microfluidically combined with droplets containing cell lysate generated with or without the protease reagent (Fig. 1B). Prior to PCR amplification, the oligonucleotides were photo-released from the hydrogel supports with UV exposure. Consistent with our earlier single-cell TaqMan reaction observations, amplification of the targeted genomic loci was substantially improved by use of a protease during cell lysis. Although similar numbers of input cells were used for both conditions, the use of protease enabled greater sequencing library DNA yields as assessed by a Bioanalyzer (Fig. 2B). Moreover, following sequencing, the average read coverage depth for the eight targets from each cell was considerably higher when protease was used in the workflow (Fig. 2C). These data demonstrate the advantage of the two-step workflow for efficient amplification across different genomic loci for targeted single-cell sequencing with barcodes.

### Targeted sequencing of AML tumor samples

**Figure 3.**
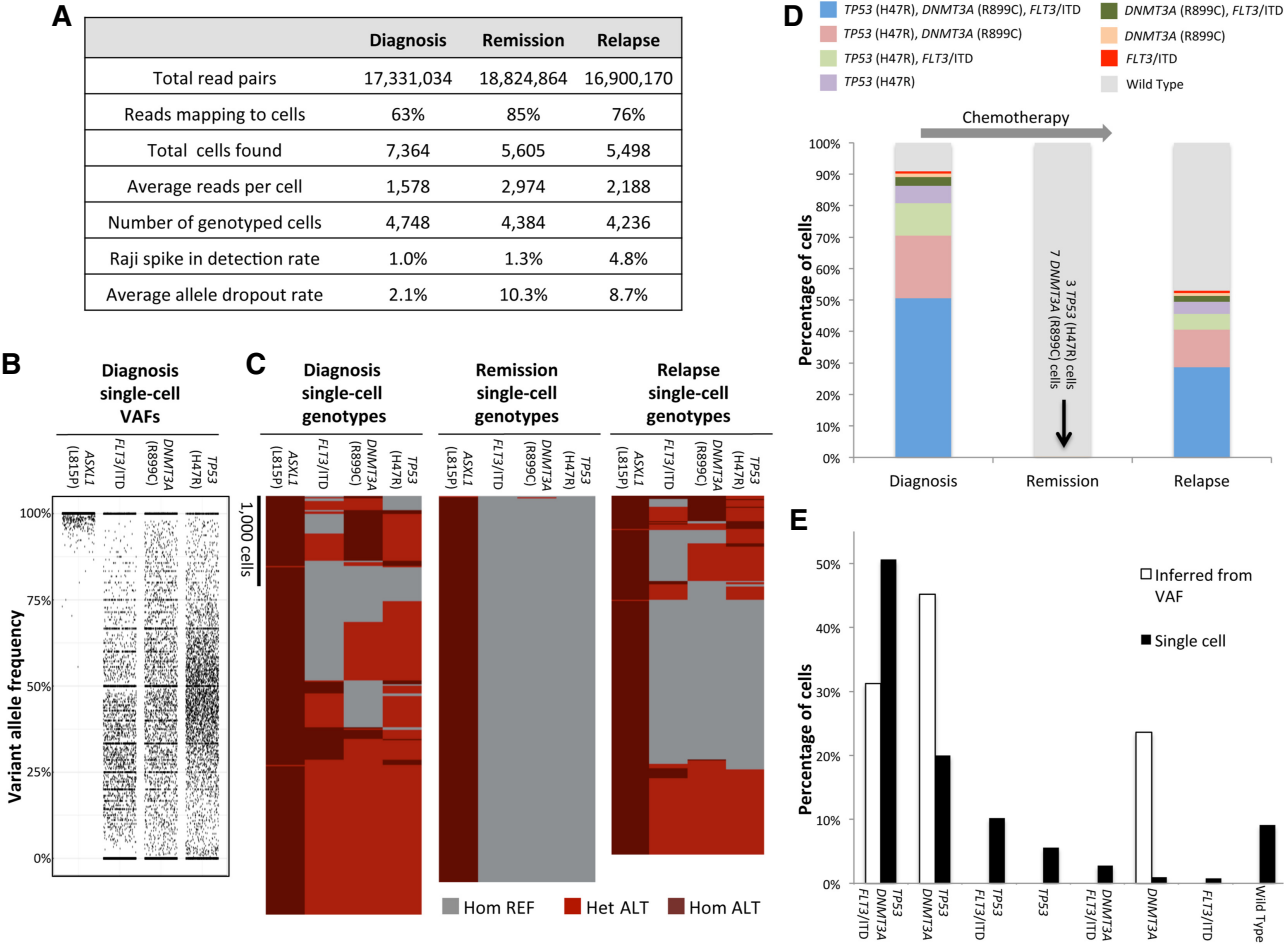
Analysis of AML clonal architecture. (*A*) Table displaying key metrics from the diagnosis, remission, and relapse single-cell DNA sequencing runs from one patient. (*B*) Diagnosis sample single-cell VAFs for each of the four nonsynonymous mutations identified for this patient. (*C*) Heat maps denoting single-cell genotypes for the three longitudinal patient samples. The presence of a heterozygous alternate (ALT) allele is shown in red. Homozygous alternate alleles are shown in dark red, and reference alleles are depicted in gray. (*D*) Clonal cell populations identified from clinical bone marrow biopsies taken at the time of diagnosis, remission, and relapse. Wild type indicates cells that had reference genome sequence for *TP53*, *DNMT3A*, and *FLT3* but were homozygous for the *ASXL1* (L815P) mutation. (*E*) Comparison of single-cell sequencing data from the diagnosis sample obtained from our workflow and a simple clonal inference of the diagnosis cell populations produced from the bulk VAFs. Nonpatient Raji cells have been removed for the analyses in *C* through *E*.

Having developed the core capability to perform targeted single-cell DNA sequencing, we next sought to apply the technology to the study of clonal heterogeneity in the context of normal karyotype AML. To provide variant allele information at clinically meaningful loci, we developed a 62-amplicon targeted panel that covers many of the 23 most commonly mutated genes associated with AML progression (Supplemental Table S1; The Cancer Genome Atlas Research Network 2013; Papaemmanuil et al. 2016). Following optimization for uniformity of amplification across the targeted loci (Supplemental Fig. S1), this panel was then used for single-cell targeted sequencing on AML patient bone marrow aspirates collected longitudinally at diagnosis, complete remission (CR), and relapse. Following thawing of frozen aspirates, the cells were quantified, and immortalized Raji cells were added to the sample to achieve an approximate 1% spike in cell population. Known heterozygous SNVs within the Raji cells served as a positive control for cell type identification and a way to assess allele dropout in the workflow. Cell suspensions were then emulsified and barcoded with our workflow prior to bulk preparation of the final sequencing libraries. Total workflow time for each sample was <2 d. MiSeq runs generating 250-bp paired-end reads were performed for each of the three samples that were barcoded. On average, 74.7% of the reads (MAPQ > 30) were associated with a cell barcode and correctly mapped to one of the 62-targeted loci (Fig. 3A). Performance of the panel across the targeted loci is shown in Supplemental Figure S2. The Raji cell spike in detection rate across the three sample runs averaged 2.4%, and the average allele dropout rate, calculated from two separate heterozygous *TP53* SNVs present in the Raji cells, was 7.0% (Fig. 3A). The allele dropout rate represents the percentage of cells within a run, averaged across the two loci, where the known heterozygous SNV was incorrectly genotyped as either homozygous wild type or homozygous mutant. To verify that the allele dropout rates are not solely dependent on those specific SNVs, we performed additional barcoding sample runs with Raji cells and calculated the dropout rates across nine variants that differ between the cell line and the reference genome (Supplemental Fig. S3). On average, the dropout rate was 8.7% ± 1.1% (SEM) across all loci for six separate experiments. To further assess the performance of our method, additional mixed cell line control experiments were performed to assess the multiplet rate, where two or more cells are assigned to the same barcode, and show that cells can be discreetly identified with the barcoding workflow (Supplemental Fig. S3).

### Single-cell variant calling and clonal analysis of AML

By using standard genotype calling algorithms (see Methods), we identified a total of 17 variant alleles for the patient in Figure 3 (Supplemental Table S2; Supplemental Fig. S4). While 13 of these variants occurred in noncoding DNA, three nonsynonymous SNVs were found in coding regions of *TP53* (H47R), *DNMT3A* (R899C), and *ASXL1* (L815P) from all three longitudinal samples (Fig. 3C,D). *ASXL1* (L815P) is a previously reported common polymorphism (dbSNP: rs6058694) and was likely present in the germline since it was found in all cells throughout the course of the disease (Schnittger et al. 2011). Additionally, a 21-bp internal tandem duplication (ITD) in *FLT3* was detected in cells from the diagnosis and relapse samples. *FLT3*/ITD alleles are found in roughly a quarter of newly diagnosed adult AML patients and are associated with poor prognosis (Kottaridis et al. 2001; Thiede et al. 2002; Papaemmanuil et al. 2016). A total of 13,368 cells (4456 cells per run average) were successfully genotyped at the four variant genomic loci (Fig. 3A–C). A comparison of the clonal populations from the diagnosis, remission, and relapse samples indicates that the patient initially achieved CR, although having 10 mutant cells may demonstrate the presence of residual disease at this time point (Fig. 3D). Despite the initial positive response to therapy, the reemergence of the clones present at diagnosis in the relapse sample indicates that it was ineffective at eradicating all of the cancer cells and, in this instance, did not dramatically remodel the initial clonal architecture of the tumor. Single-cell sequencing of additional cells from the remission sample would likely be required to test this hypothesis and identify additional residual clones during remission.

To assess the performance of our single-cell approach relative to conventional next-generation sequencing (see Methods), we obtained bulk variant allele frequencies (VAFs) for the relevant mutations in two of the biopsy samples. The bulk VAFs were comparable to the VAFs acquired from our single-cell sequencing workflow when the barcode identifiers are removed and the reads are analyzed in aggregate (Supplemental Fig. S5). We next used the bulk sample VAFs to infer clonal architecture and compare it to the clonal populations obtained with our single-cell sequencing approach. The simplest model of inferred clonality predicts a significant *DNMT3A* (R899C) single-mutant population indicative of founder mutation status (Fig. 3E). The single-cell sequencing data do not support this model as only a relatively small *DNMT3A* single-mutant population is observed and this population is at a frequency that can be explained by allele dropout. In contrast, our results suggest that the SNV in *TP53* could be the founding mutation since the size of the *TP53* (H47R) single-mutant clone is larger than what would be expected from allele dropout. Our single-cell approach also unambiguously identified the *TP53*, *DNMT3A* and *FLT3*/ITD triple-mutant population as the most abundant neoplastic cell type in the diagnosis and relapse samples (Fig. 3D). Moreover, the identification of this clone strongly supports a model where the mutations were serially acquired during the progression of the disease.

### Clonal remodeling of an AML tumor at relapse

**Figure 4.**
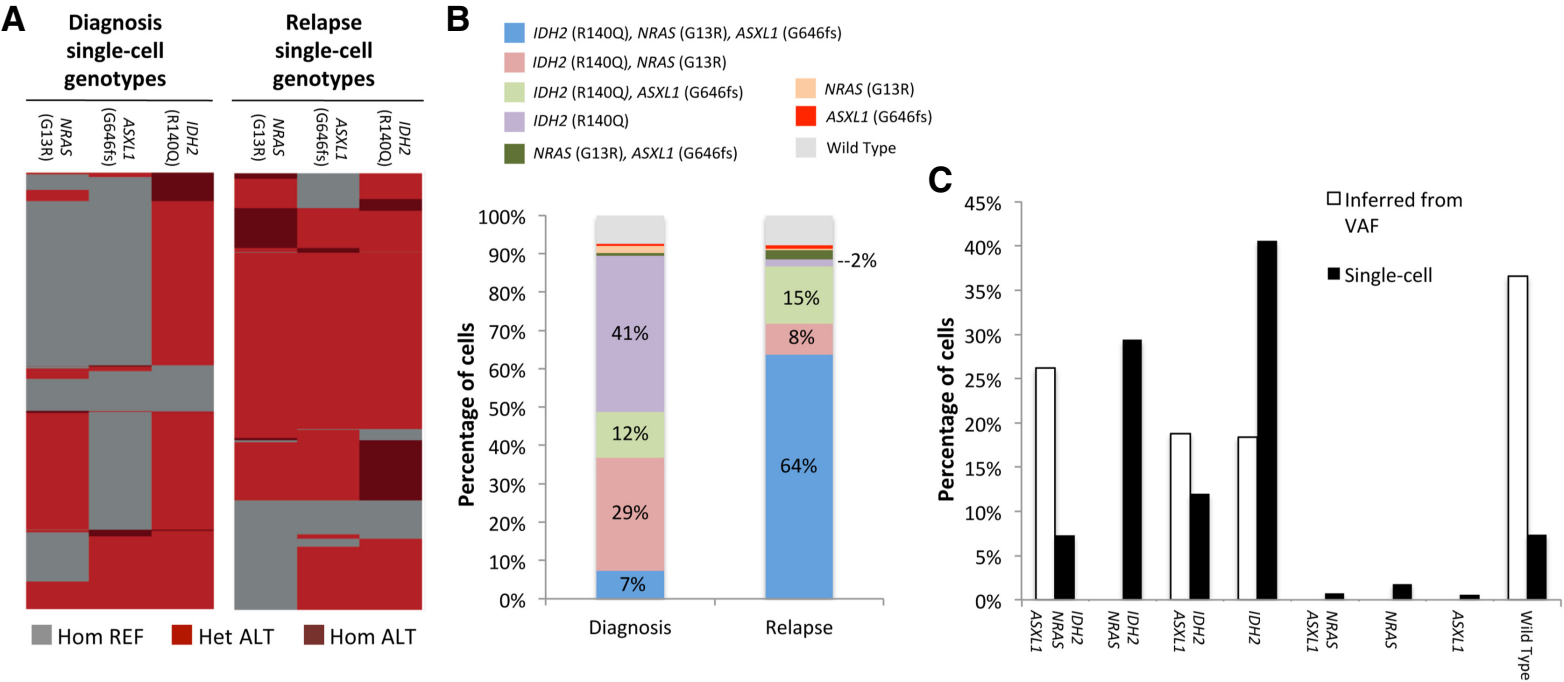
Clonal remodeling of an AML tumor. (*A*) Heat maps denoting single-cell genotypes for the diagnosis and relapse samples. The presence of a heterozygous alternate (ALT) allele is shown in red. Homozygous alternate alleles are shown in dark red, and reference alleles are depicted in gray. (*B*) Clinical bone marrow biopsies taken at the time of diagnosis and relapse show substantial changes in clonal distribution with single-cell sequencing. Wild type indicates cells that had reference genome sequence for *IDH2*, *ASXL1*, and *NRAS*. (*C*) Comparison of single-cell sequencing data from the diagnosis sample obtained from our workflow and a simple clonal inference of the diagnosis cell populations produced from the bulk VAFs. Nonpatient Raji cells have been removed from these data sets.

To further investigate the ability of high-throughput single-cell sequencing to accurately characterize the clonal architecture of tumors, we analyzed diagnosis and relapse bone marrow aspirate samples from a second patient with normal karyotype AML. By using our variant calling approach, 20 genetic variants were confidently identified, and of those, five were nonsynonymous mutations (Supplemental Table S3). *ASXL1* (L815P) was again identified as a nonsynonymous polymorphism in this patient, further validating its status as a common variant. We focused subsequent analysis on the disease-relevant mutations that were also identified in bulk sequencing of the diagnosis sample: *IDH2* (R140Q), *NRAS* (G13R), and *ASXL1* (G646fs). A total of 2850 cells were accurately genotyped at these three variant genomic loci (Fig. 4A). The Raji cell spike in detection rate for these runs averaged 1.5%, and the average allele dropout rate, calculated from the Raji cells, was 8.5%.

By using the genotype calls from individual cells, the clonal composition of both diagnosis and relapse tumors was reconstructed, as shown in Figure 4B. There were a number of clones that significantly expanded or contracted during the course of the disease. The most dramatic of these changes were an expansion of the triple-mutant *IDH2* (R140Q) *NRAS* (G13R) *ASXL1* (G646fs) clone from 7% at diagnosis to 64% at relapse and a reduction in the *IDH2* (R140Q) single-mutant population from 41% at diagnosis to 2% at relapse. These changes cannot be explained by technical noise alone since the allele dropout rates were almost identical between the two sample runs (diagnosis = 8.0%, relapse = 9.0%). Additionally, if these clonal distribution changes were systematic and technical in nature, we would have expected to see similar changes in the first patient, where the diagnosis and relapse samples were almost identical in composition. One possible factor contributing to the clonal evolution seen in the second patient is the extended 3-yr time period that elapsed between remission and relapse.

Lastly, we used VAFs generated from bulk sequencing to infer clonal architecture of the diagnosis sample. This inferred architecture differed substantially from the single-cell sequencing derived populations (Fig. 4C). Notably, the *IDH2* (R140Q) *NRAS* (G13R) clone was not predicted from the bulk sequencing VAFs, yet it represented 29% of the diagnosis tumor defined by single-cell DNA sequencing. The *IDH2* (R140Q) *NRAS* (G13R) population shrinks to 8% of the tumor at the same relapse time point at which the triple-mutant *IDH2* (R140Q) *NRAS* (G13R) *ASXL1* (G646fs) clone expands to 64%. Consequently, it is not likely that the *IDH2* (R140Q) *NRAS* (G13R) clone is a result of *ASXL1* (G646fs) allele dropout in the triple-mutant cells given the much greater size of the triple-mutant cell population at relapse and similar observed allele dropout rates. This provides another clear example of the high-resolution clonal architecture uncovered by single-cell DNA analysis that is missed with bulk sequencing data.

## Discussion

Our method enables rapid and cost-effective targeted genome sequencing of thousands of tumor cells in parallel—something that is not feasible with existing technologies. Previously, single-cell DNA analysis was most commonly performed with laboratory-developed approaches relying upon FACS sorting to first isolate single-cells in 96- or 384-well plates (e.g., Paguirigan et al. 2015). Not only are these approaches laborious and slow, but they also utilize significant amounts of reagent to generate sequence information. The workflow we developed can generate sequence ready libraries in <2 d and, through the use of picoliter volume droplets, consumes minimal reagent to barcode genomic DNA. These key features significantly lower the barriers for performing single-cell DNA sequencing and promise to make high-resolution analysis of clonal architecture within tumors routine. Although the panel used in this study comprised 62 amplicons, we are currently developing capability for increased multiplexing. This should enable a more comprehensive analysis of AML and reduce the genome coverage deficit between our method and well-based single-cell approaches that perform whole-exome or -genome sequencing.

We anticipate that applying our approach to the study of larger AML patient populations will lead to correlations between clonal heterogeneity and clinical outcomes. We have already observed a marked difference in the pattern of clonal evolution between the two patients analyzed in this study. Patient 1 (Fig. 3) had an almost identical clonal architecture in both diagnosis and relapse, whereas patient 2 (Fig. 4) showed a significant shift in the clonal populations present at relapse. While the data from two patients do not allow us to draw a definitive conclusion, it is conceivable that the difference in treatment and remission duration may be associated with the distinct patterns of clonal evolution. Patient 1 had a short-lived remission of 3 mo, whereas patient 2 had 3 yr of remission and received low-dose chemotherapy for a prolonged period. The latter might have contributed to the selection of chemotherapy-resistant clones that expanded at relapse. A study relying on a larger number of patients with variable remission duration and treatment history may provide a stronger association between the pattern of clonal evolution and clinical history. Another correlation that could be made is the detection of residual disease with single-cell sequencing during remission and risk for disease relapse. We show that our technology was able to detect as few as three mutation-harboring cells out of about 4000 genotyped cells from remission biopsies. The ability to detect specific residual tumor subclones in this fashion could complement existing minimal residual disease monitoring strategies by identifying specific subclones with co-occurring mutations that may be more prognostic for the disease than bulk molecular measurements alone (Chen and Wood 2017). Due to the scalability of droplet-based microfluidics, improving the throughput and subclone sensitivity with our method is straightforward. Single-cell DNA sequencing at high-throughput could one day displace multiparametric flow cytometry as the standard for minimal residual disease monitoring.

The use of targeted single-cell sequencing for accurate analysis of clonal architecture may also uncover key features related to the genesis and evolution of AML tumors. We show significant differences between bulk sequencing results and actual subclones present as revealed by single-cell genotyping. In the first patient we analyzed (Fig. 3), we show that the *DNMT3A* single-mutant subclone was present at only 1.7%, when the bulk sequencing predicted it to comprise 23.6% of the tumor at diagnosis. This discrepancy casts doubt on the founder mutation status of *DNMT3A* in this patient. Similarly, in the second patient (Fig. 4), inference of clones from the bulk sequencing data failed to predict the presence of the *IDH2* (R140Q), *NRAS* (G13R) double-mutant clone comprising ∼30% of the total tumor population. We measured the allele dropout rate with multiple control loci in all of our single-cell sequencing experiments and determined that this technical issue is not sufficient to explain the differences we observed with bulk sequencing predictions. Nevertheless, our current observations are based on a small number of patients and a single approach to bulk sequencing. Studies with other bulk sequencing panels and additional patients will be required to confirm our initial observations and demonstrate the clinical utility of the approach we described in this report.

Despite the improved clonal resolution provided by our method, some clonal populations are likely technical in nature and probably a consequence of allele dropout. For example, in the patient 2 relapse sample (Fig. 4B), there are multiple clones present at <2.5%, and three of them are single-mutant genotypes for each of the pathogenic variants that were identified. Not only can these clones be statistically explained with allele dropout, but they also do not fit with a model of serial mutation acquisition predicted by the presence of the *NRAS*, *ASXL1*, and *IDH2* triple-mutant clone comprising 64% of the tumor at this time point. Consequently, observed allele dropout rates as well as the tumor biology and evolution must all be taken into account when interpreting clonal populations identified with our single-cell approach.

Although we focused on AML in this study, our method should be applicable to other cancer cell types and profiling of solid tumors that have been dissociated into single-cell suspensions. Correspondingly, the targeted sequencing panel used in this study can be readily changed to target genomic loci relevant to different cancer types. This capability is poised to complement an increased scientific appreciation of the role that genetic heterogeneity plays in the progression of many cancers as well as a desire by clinicians to make precision medicine a widespread reality.

## Methods

### Cell and patient samples

All patient samples were collected under an IRB-approved protocol and patients signed the consent for sample collection and analysis. The protocol adhered to the Declaration of Helsinki. The clinical AML samples presented in Figure 3 were obtained from a 66-yr-old man who was diagnosed with AML, French-American-British (FAB) classification M5. A pretreatment diagnostic bone marrow biopsy showed 80% myeloblast, and cytogenetic analysis showed normal male karyotype. He received an induction chemotherapy consisting of fludarabine, cytarabine, and idarubicin. Day 28 bone marrow aspiration showed morphological CR. He received an additional two cycles of consolidation therapy with the same combination, but ∼3 mo after achieving CR, his AML relapsed with 48% blast. He was subsequently treated with azacitidine and sorafenib chemotherapy and achieved a second CR. He then underwent allogeneic stem cell transplant from his matched sibling, but ∼2 mo after transplant, his disease relapsed. He was subsequently treated with multiple salvage therapies, but he passed away from leukemia progression ∼2 yr from his original diagnosis. Bone marrow from the original diagnosis, first CR, and first relapse was analyzed.

The second patient analyzed and presented in Figure 4 was a 65-yr-old man diagnosed with AML having myelodysplastic changes and 44% myeloblast, and cytogenetic analysis showed a normal karyotype. Bulk sequencing VAFs for *IDH2* (R140Q), *NRAS* (G13R), and *ASXL1* (G646fs) were 31.7%, 13.1%, and 22.5%, respectively, in the diagnosis sample. The patient received induction chemotherapy with cladribine and cytarabine and achieved CR at day 28. He completed consolidation therapy for 18 cycles and then received maintenance therapy with decitabine for additional 12 cycles. Approximately 3 yr after achieving CR, patient relapsed with 20% blast. Tumor cell samples were not available for the remission time point of this patient.

Raji B-lymphocyte cells were cultured in complete media (RPMI 1640 with 10% fetal bovine serum [FBS], 100 U/mL penicillin, and 100 µg/mL streptomycin) at 37°C with 5% CO_2_. Cells were pelleted at 400*g* for 4 min and washed once with HBSS and resuspended in PBS that was density matched with OptiPrep (Sigma-Aldrich) prior to encapsulation in microfluidic droplets. Frozen bone marrow aspirates were thawed at the time of cell encapsulation and resuspended in 5 mL of FBS on ice, followed by a single wash with PBS. All cell samples were quantified prior to encapsulation by combining 5 µL aliquots of cell suspension with an equal amount of trypan blue, loaded on chamber slides, and counted with the Countess automated cell counter (Thermo Fisher). A total of 200,000–250,000 bone marrow aspirate–derived cells were used in each of the sample barcoding runs. The Raji cells were added to the bone marrow cell samples to achieve a ∼1% final spike-in concentration.

### Fabrication and operation of microfluidic devices

We performed the microfluidic droplet handling on devices made from polydimethylsiloxane (PDMS) molds bonded to glass slides; the device channels were treated with Aquapel to make them hydrophobic. The PDMS molds were formed from silicon wafer masters with photolithographically patterned SU-8 (MicroChem) on them. We operated the devices primarily with syringe pumps (NewEra), which drove cell suspensions, reagents, and fluorinated oils (Novec 7500 and FC-40) with 2%–5% PEG-PFPE block-copolymer surfactant into the devices through polyethylene tubing (Holtze et al. 2008). Merger of the cell lysate containing droplets with the PCR reagent/barcode bead droplets was performed using a microfluidic electrode (Sciambi and Abate 2014).

### Generation of barcode containing beads

Barcoded hydrogel beads were made as previously reported (Klein et al. 2015). Briefly, a monomeric acrylamide solution and an acrydite-modified oligonucleotide were emulsified on a dropmaker with oil containing TEMED. The TEMED initiates polymerization of the acrylamide resulting in highly uniform beads. The incorporated oligonucleotide was then used as a base on which different split-and-pool–generated combinations of barcodes were sequentially added with isothermal extension. Targeted gene-specific primers were phosphorylated and ligated to the 5′ end of the hydrogel attached oligonucleotides. ExoI was used to digest nonligated barcode oligonucleotides that could otherwise interfere with the PCR reactions. Because the acrydite oligo also has a photocleavable linker (required for droplet PCR), barcoded oligonucleotide generation could be measured. We were able to convert ∼45% of the base acrydite oligonucleotide into full-length barcode with gene specific primers attached. Single-bead sequencing of beads from individual bead lots was also performed to verify quality of this reagent.

### Cell encapsulation and droplet PCR

Following density matching, cell suspensions were loaded into 1-mL syringes and coflowed with an equal volume of lysis buffer (100 mM Tris at pH 8.0, 0.5% IGEPAL, proteinase K 1.0 mg/mL) to prevent premature lysing of cells (Eastburn et al. 2013). The resultant emulsions were then incubated for 16–20 h at 37°C prior to heat inactivation of the protease.

Droplet PCR reactions consisted of 1× platinum multiplex PCR master mix (Thermo Fisher Scientific), supplemented with 0.2 mg/mL RNAse A. Prior to thermocycling, the PCR emulsions containing the barcode carrying hydrogel beads were exposed to UV light for 8 min to release the oligonucleotides (Zilionis et al. 2017). Droplet PCR reactions were thermocycled with the following conditions: 10 min at 95°C; 25 cycles of 30 sec at 95°C, 10 sec at 72°C, 4 min at 60°C, and 30 sec at 72°C; and a final step of 2 min at 72°C. Single-cell TaqMan reactions targeting the *SRY* locus were performed as previously described (Eastburn et al. 2014).

### DNA recovery and sequencing library preparation

Following thermocycling, emulsions were broken using perfluoro-1-octanol, and the aqueous fraction was diluted in water. The aqueous fraction was then collected and centrifuged prior to DNA purification using 0.63× of SPRI beads (Beckman Coulter). Sample indexes and Illumina adapter sequences were then added via a 10 cycle PCR reaction with 1× Phusion High-Fidelity PCR Master Mix. A second 0.63× SPRI purification was then performed on the completed PCR reactions and samples were eluted in 10 µL of water. Following the second PCR and SPRI purification, full-length amplicons are ready for quantification and sequencing; no further fragmentation or library preparation steps are necessary. Libraries were analyzed on a DNA 1000 assay chip with a Bioanalyzer (Agilent Technologies) and sequenced on an Illumina MiSeq with either 150-bp or 250-bp paired end multiplexed runs. A single sequencing run was performed for each barcoded single-cell library prepared with our microfluidic workflow. A 5% ratio of PhiX DNA was used in the sequencing runs.

### Analysis of next-generation sequencing data

Sequenced reads were trimmed for adapter sequences (Cutadapt) (Martin 2011; Bolger et al. 2014) and aligned to the hg19 human genome using BWA-MEM (Langmead et al. 2009; Kim et al. 2013) after extracting barcode information. Following mapping, on target sequences were selected using standard bioinformatics tools (SAMtools) (Li et al. 2009), and barcode sequences were error-corrected based on a white list of known sequences. The reads that were unable to be correctly mapped to cells comprised a mix of barcodes that were not able to be error-corrected to a known barcode, lacked an insert sequence between the gene-specific primer sequences, or mapped to off target loci. The number of cells present in each tube was determined based on curve fitting a plot of number of reads assigned to each barcode versus barcodes ranked in decreasing order, similar to what was described previously (Supplemental Fig. S3; Macosko et al. 2015). The total number of cells identified in this manner for a given sample run are presented in Figure 3A as “total cells found.” A subset of these cells was then identified that had sufficient sequence coverage depth to call genotypes at the nonsynonymous variant positions identified in *TP53*, *ASXL1*, *FLT3,* and *DNMT3A.* This subset of cells is presented as “number of genotyped cells” in Figure 3A.

GATK 3.7(McKenna et al. 2010) was used to genotype diagnosis samples with a joint-calling approach. The quality score of known Raji cell mutations was used to set a minimum threshold for variant calling in patient cells. For the first patient (Fig. 3), the presence of these variants as well as the potential *FLT3*/ITD were called at a single-cell level across the three samples using FreeBayes (Garrison and Marth 2012). Genotype cluster analysis was performed using Heatmap3 for R (Zhao et al. 2014). The nonpatient Raji cell spike in populations were removed for these analyses.

### Bulk sequencing using capture targeted sequencing

We designed a SureSelect custom panel of 295 genes (Agilent Technologies) that are recurrently mutated in hematologic malignancies (Supplemental Table S4). Extracted genomic DNA from bone marrow aspirates was fragmented and bait-captured according to manufacturer protocols. Captured DNA libraries were then sequenced using a HiSeq 2000 sequencer (Illumina) with 76-bp paired-end reads.

## Data access

The sequencing data from this study have been submitted to the NCBI database of Genotypes and Phenotypes (dbGaP; https://www.ncbi.nlm.nih.gov/gap) under accession number phs001627.v1.p1.

## Competing interest statement

M.P., A.S., S.T., R.D.-D., K.G., J.J., T.X.C., J.A.G., W.O., K.W.J., and D.J.E. are employees and shareholders of Mission Bio, Inc.

## Supplementary Material

Supplemental Material
