# Supplementary material for: High-throughput single-cell DNA sequencing of acute myeloid leukemia tumors with droplet microfluidics
Source: Genome Res. 2018 Sep;28(9):1345–52. doi: 10.1101/gr.232272.117 (PMC6120635; doi:10.1101/gr.232272.117)
Supplement: Supplemental Material [file supp_28_9_1345__index.html]

High-throughput single-cell DNA sequencing of acute myeloid leukemia tumors with droplet microfluidics — Supplemental Material 

# High-throughput single-cell DNA sequencing of acute myeloid leukemia tumors with droplet microfluidics

## Supplemental Material

- Supplemental\_Fig\_S1.pdf
- Supplemental\_Fig\_S2.pdf
- Supplemental\_Fig\_S3.pdf
- Supplemental\_Fig\_S4.pdf
- Supplemental\_Fig\_S5.pdf
- Supplemental\_Table\_S1.pdf
- Supplemental\_Table\_S2.pdf
- Supplemental\_Table\_S3.pdf
- Supplemental\_Table\_S4.pdf
