# Supplementary material for: High-throughput single-cell DNA sequencing of acute myeloid leukemia tumors with droplet microfluidics
Source: Genome Res. 2018 Sep;28(9):1345–52. doi: 10.1101/gr.232272.117 (PMC6120635; doi:10.1101/gr.232272.117)
Supplement: Supplemental Material [file supp_gr.232272.117_Supplemental_Fig_S1.pdf]

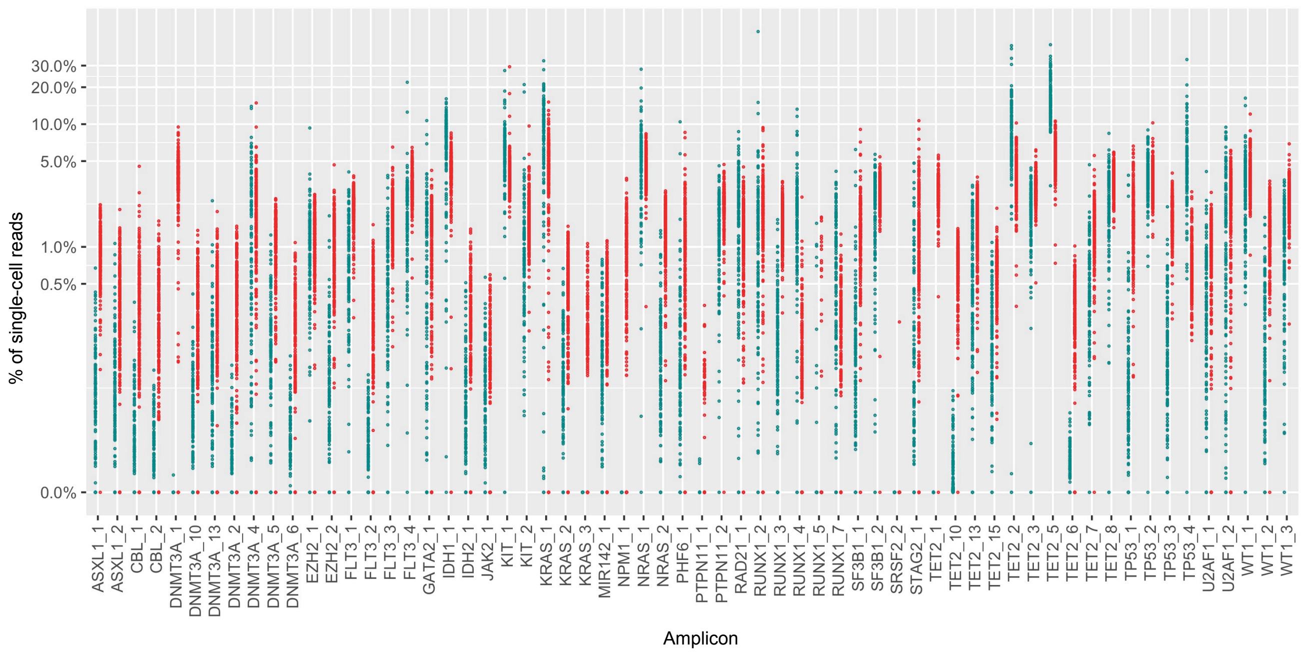

Supplemental Figure S1. *Uniformity of amplification across the 62 amplicon AML panel.* The percentage of total reads per cell is plotted for each of the 62 amplicons in the targeted AML panel. Each point in the plot represents the data from a single-cell. Blue colored data points were generated for droplet amplification conditions prior to uniformity optimization. Red data points were generated following optimization of thermocycling conditions and reaction components. For each of the two separate conditions the same 250 cells are shown across all of the different sequenced loci.
