# Supplementary material for: High-throughput single-cell DNA sequencing of acute myeloid leukemia tumors with droplet microfluidics
Source: Genome Res. 2018 Sep;28(9):1345–52. doi: 10.1101/gr.232272.117 (PMC6120635; doi:10.1101/gr.232272.117)
Supplement: Supplemental Material [file supp_gr.232272.117_Supplemental_Fig_S2.pdf]

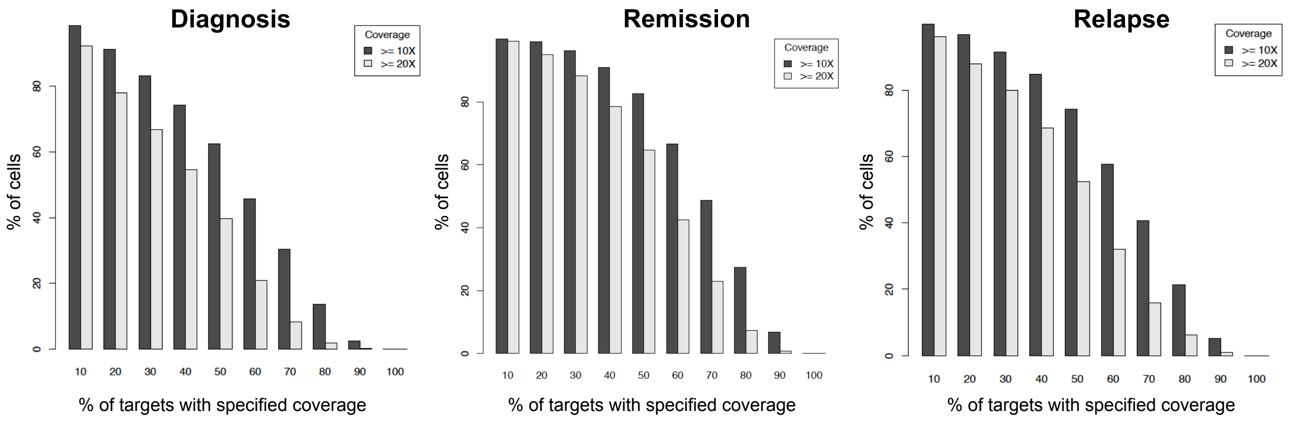


**Supplemental Figure S2.** *Single-cell targeted panel amplification performance.* Histograms showing the percentage of the 62 target amplicons present at either 10X (dark gray) or 20X (light gray) coverage across the cells identified in our bioinformatics pipeline. Data is shown for single-cell sequencing of the diagnosis, remission and relapse samples.
