# Supplementary material for: High-throughput single-cell DNA sequencing of acute myeloid leukemia tumors with droplet microfluidics
Source: Genome Res. 2018 Sep;28(9):1345–52. doi: 10.1101/gr.232272.117 (PMC6120635; doi:10.1101/gr.232272.117)
Supplement: Supplemental Material [file supp_gr.232272.117_Supplemental_Fig_S3.pdf]

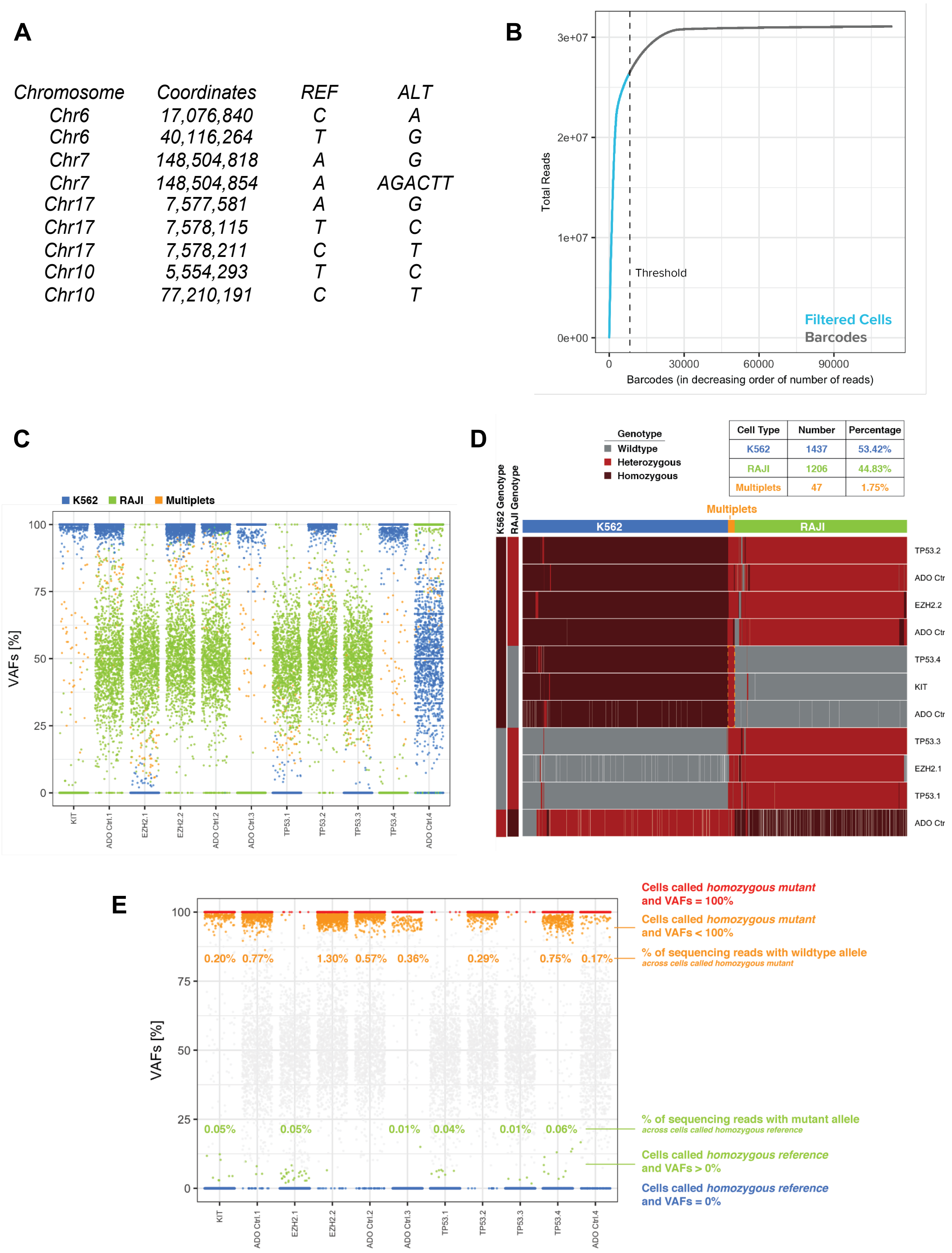

Supplemental Figure S3. *Cell line genotyping experiments.* (A) Chromosomal positions and sequences of variants used to calculate allele drop out rates for Raji cells. (B) Roughly equal numbers of Raji or K562 cells were combined and run through the single-cell barcoding workflow. Cumulative plot for the mixed cell line experiment showing the

number of reads associated with cell identifying barcodes following sequencing. Barcodes identified as cells (Filtered Cells) are shown to the left of the vertical dashed threshold line. (C) Single-cell variant allele frequency (VAF) distributions across cell-line distinguishing variants. Dots represent individual cells. Cells are color-coded (blue, green or orange) according to where they clustered on the heat map in (D). (D) Heat maps display single cell genotype calls at 11 variant loci that differentiate each cell line. The expected genotype for each cell type is shown on the left side of the heat map. The multiplet column (marked with orange) indicates cells that displayed genotypes consistent with a mixture of the two cell lines. (E) Quantification of variant mixing between cell types. Cells colored in red are called homozygous mutant with VAFs at hundred percent. Cells colored in blue are called homozygous reference with VAFs at zero percent. Cells colored in orange and green are called homozygous mutant or reference, respectively with VAFs other than hundred or zero percent. A small percentage of reads, in only a fraction of the cells, are of the alternate cell genotype. This level of alternate read mixing is likely to minimally impact the correct genotype call at a given variant position. The mixed cell line experiments performed in this figure were carried out with a modified version of the 62 amplicon AML panel. This modified panel is the commercially available Tapestri AML panel from Mission Bio, Inc.
