# Supplementary material for: High-throughput single-cell DNA sequencing of acute myeloid leukemia tumors with droplet microfluidics
Source: Genome Res. 2018 Sep;28(9):1345–52. doi: 10.1101/gr.232272.117 (PMC6120635; doi:10.1101/gr.232272.117)
Supplement: Supplemental Material [file supp_gr.232272.117_Supplemental_Fig_S4.pdf]

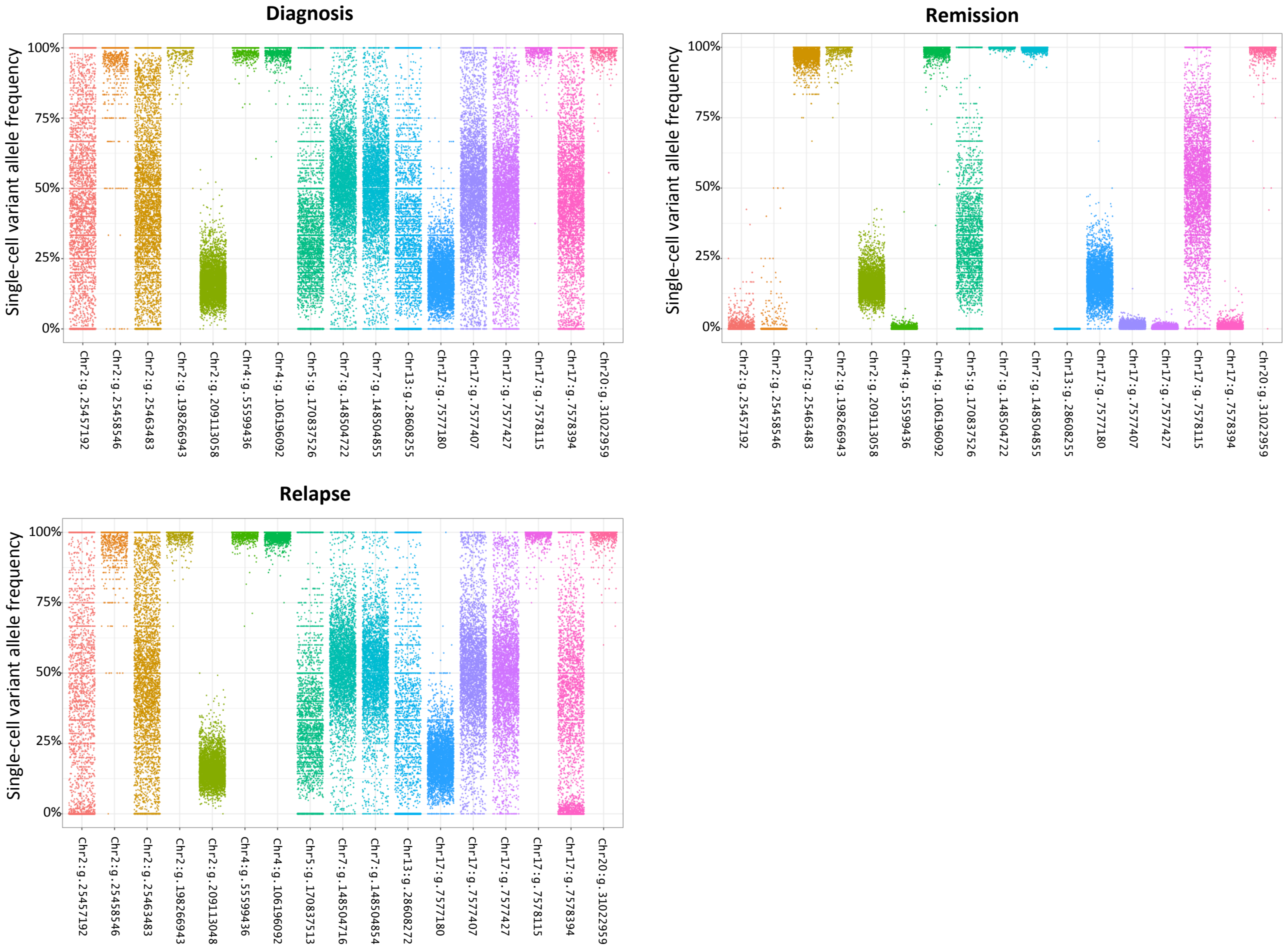

Supplemental Figure S4. *Single-cell variant allele frequencies.* Single cell VAFs are plotted for all 17 of the identified genetic variants found in the diagnosis, complete remission and relapse samples.
