# Supplementary material for: High-throughput single-cell DNA sequencing of acute myeloid leukemia tumors with droplet microfluidics
Source: Genome Res. 2018 Sep;28(9):1345–52. doi: 10.1101/gr.232272.117 (PMC6120635; doi:10.1101/gr.232272.117)
Supplement: Supplemental Material [file supp_gr.232272.117_Supplemental_Fig_S5.pdf]

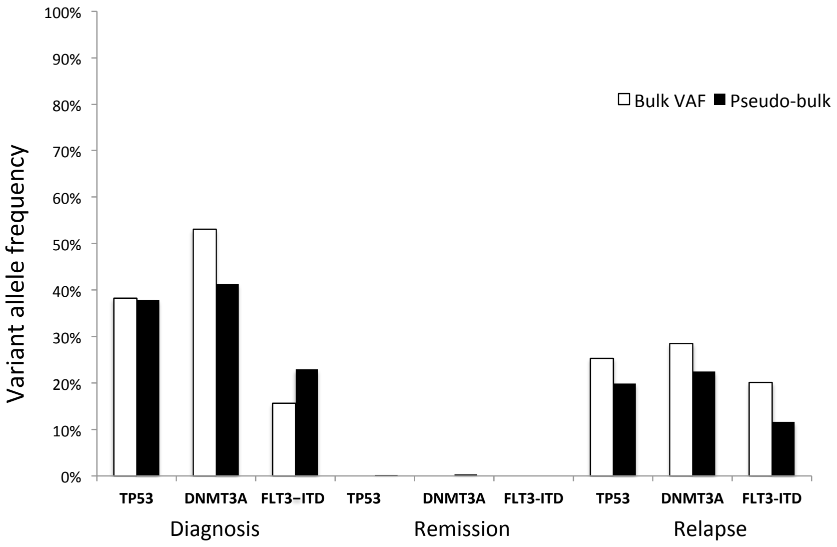

Supplemental Figure S5. *Comparison of bulk and pseudo bulk VAFs*. Variant allele frequencies for mutations in *TP53*, *DNMT3A* and *FLT3* are displayed for diagnosis, remission and relapse samples. VAFs obtained from traditional bulk next generation sequencing are displayed as open bars. The black bars represent the VAFs obtained from our single-cell sequencing workflow; however, the barcode identifiers have been removed and the reads have been analyzed as a single population to give a "pseudo-bulk" frequency. Bulk sequencing was not performed on the remission sample; therefore, VAF data is not available for this timepoint.
