# Supplementary material for: High-throughput single-cell DNA sequencing of acute myeloid leukemia tumors with droplet microfluidics
Source: Genome Res. 2018 Sep;28(9):1345–52. doi: 10.1101/gr.232272.117 (PMC6120635; doi:10.1101/gr.232272.117)
Supplement: Supplemental Material [file supp_gr.232272.117_Supplemental_Table_S1.pdf]

Chromosome	Gene	fwd	rev	Amplicon name
Chr1	**NRAS**	ACAACCTAAAACCAACTCTTCCCATAATT	TGGTGAAACCTGTTTGTTGGACAT	NRAS_1
Chr1	**NRAS**	CACGTTAAGCTTATTGCATAACTGAATGT	GGTTCTTGCTGGTGTGAAATGAC	NRAS_2
Chr11	**WT1**	TCCTTCTCTCAACTGAGTCTAAACCTT	CTCACTGTGCCCACATTGTTAG	WT1_2
Chr11	**WT1**	CTAAACACATGGCTGACTCTCTCA	TAGTAGGAGAGGTTGCCTTTAATGAGAT	WT1_1
Chr11	**WT1**	GCCTGGAAAAGGAGCTCTTGAA	TCAAGACCTACGTGAATGTTCACATG	WT1_3
Chr11	**CBL**	AGGAAACAAGTCTTCACTTTTTCTGTTAAC	ACCGAATTTTCCAAGGTTATTACATAGCT	CBL_2
Chr11	**CBL**	TTCAGATGCATCTGTTACTATCTTTTGCT	GATTTTGCCAGTCTCCTAAACTGC	CBL_1
Chr12	**KRAS**	TCCTCATGTACTGGTCCCTCAT	CGTCATCTTTGGAGCAGGAACAAT	KRAS_2
Chr12	**KRAS**	AAAGAATGGTCCTGCACCAGTAA	AAAGGTGAGTTTGTATTAAAAGGTACTGGT	KRAS_1
Chr12	**KRAS**	CAGATCTGTATTTATTTCAGTGTTACTTACCT	GACTCTGAAGATGTACCTATGGTCCTA	KRAS_3
Chr12	**PTPN11**	GCCTCCCTTTCCAATGGACTAT	GCAGCAGACTTTGTGGTCACTAAA	PTPN11_2
Chr12	**PTPN11**	CCACTAAAAGTTGTGCATTAAACAACTTCA	CTAGCAAGAGAATGAGAATCCGCAT	PTPN11_1
Chr13	**FLT3**	GCAGACTGCTGTGAGGGTTTTT	CTCTGGTGTCATTCTTGACAGTGT	FLT3_1
Chr13	**FLT3**	GAGTGCTCAGTGTCTAATTCCACTT	ACAGAAAAAGCAGACAGCTCTGAAA	FLT3_3
Chr13	**FLT3**	TCCTAGTACCTTCCCTGCAAAGA	TGCCTATTCCTAACTGACTCATCATTTC	FLT3_2
Chr13	**FLT3**	ACACTGACCCTATACTCTCCTGTAAAA	CACAGAAGGAGTCTGGAATAGAAAGG	FLT3_4
Chr15	**IDH2**	CCACAAAGTCTGTGGCCTTGTA	CCTCACAGAGTTCAAGCTGAAGAAG	IDH2_1
Chr17	**TP53**	GGAAAGAGGCAAGGAAAGGTGATA	GACCTGATTTCCTTACTGCCTCTT	TP53_4
Chr17	**TP53**	TGTGATGAGAGGTGGATGGGTA	CCTCATCTTGGGCCTGTGTTAT	TP53_2
Chr17	**TP53**	GGGTTATAGGGAGGTCAAATAAGCA	GGCCTCTGATTCCTCACTGATTG	TP53_1
Chr17	**TP53**	CTGCTCACCATCGCTATCTGAG	TGCCGTCTTCCAGTTGCTTTAT	TP53_3
Chr17	**MIR142**	CGAAGCCCACAGTACACTCATC	GGATCTTAGGAAGCCACAAGGA	MIR142_1
Chr17	**SRSF2**	CCTCAGCCCCGTTTACCT	CTTCGTTCGCTTTCACGAC	SRSF2_2
Chr2	**DNMT3A**	TTTGTGTCGCTACCTCAGTTTG	GGTCCTGCTGTGTGGTTAGAC	DNMT3A_10
Chr2	**DNMT3A**	CCAGGACGTTTGTGGAAAACAAG	TGGTGAATGAATTCGTGCTTATTCCT	DNMT3A_6
Chr2	**DNMT3A**	CAGAGCAGCTAGTCATTCAGCA	TCTTCAAACCGTCTCCTGTTTT	DNMT3A_13
Chr2	**DNMT3A**	CAGCTCCACAATGCAGATGAGA	CCAGCTGATGGCTTTCTCTTCC	DNMT3A_2
Chr2	**DNMT3A**	CTGTCCCAGGGCAGAAATATCC	CAAAGGACTTCTTTGCCAAGTTCAC	DNMT3A_5
Chr2	**DNMT3A**	GGACTGCATACGTTTCCACTTC	CATCTGACCTGTTGTGCTCACT	DNMT3A_1
Chr2	**DNMT3A**	CCCACAACTTACCACTGAGAATTTG	GCCTCGTGACCACTGTGTAATG	DNMT3A_4
Chr2	**SF3B1**	CTTCCATAAAGGCTTTAACACAGAATCAA	GCTATGGTTCATGTTTTGCTTTTACCTAAT	SF3B1_1
Chr2	**SF3B1**	TGTGTGTGTACCTCTAGTCCCAA	GTGTGCAAAAGCAAGAAGTCCT	SF3B1_2
Chr2	**IDH1**	AATGTGTTGAGATGGACGCCTATT	CTTGTGAGTGGATGGGTAAAACCTAT	IDH1_1
Chr20	**ASXL1**	CAGGACCCTCGCAGACATTAAA	GGCAGTAGTTGTGTTCGCTGTA	ASXL1_1
Chr20	**ASXL1**	CCTCAGTAGCTGAGAGATTAGTGGA	TCTGTTCTGCAGGCAATCAGTC	ASXL1_2
Chr21	**RUNX1**	CGACATGCCGATGCCGAT	CCCATCCTCCTAGGCGGTATC	RUNX1_5
Chr21	**RUNX1**	CATGGGACTCAGAGTAGAGATAGGT	CGTGGTCCTACGATCAGTCCTA	RUNX1_2
Chr21	**RUNX1**	AGTGGGCTCCATCTGGTACTTA	CCACAATAGGACATCGGCAGAAA	RUNX1_3
Chr21	**RUNX1**	CTCAGTGCACAGAAACAAGCTT	CCATCACTGTCTTCACAAACCCA	RUNX1_4
Chr21	**RUNX1**	AATTTTGAAATGTGGGTTTGTTG	GTCCTTTGACTGGTGTTTAGGTG	RUNX1_7
Chr21	**U2AF1**	GGTGGGTTGGAAGGAGACATTT	AGTCTTATTAAAGCGTGGATGGCAA	U2AF1_1
Chr21	**U2AF1**	AGTCGATCACCTGCCTCACTAT	GCTCTCATTTTCCCTTACAGAGTCAAC	U2AF1_2
Chr3	**GATA2**	AGTCTTCGCTTGGGCTTGAT	GGACTCCCTCCCGAGAACTT	GATA2_1
Chr4	**KIT**	CCTCCTTGTACCTTCCACTCCTT	CTCAGTTCCTGGACAAAAATACCAATCTAT	KIT_2
Chr4	**KIT**	AAATGGTTTTCTTTTCTCCTCCAACCTA	CTAAAATGTGTGATATCCCTAGACAGGATT	KIT_1
Chr4	**TET2**	ACAAGACCAATGTCAGAACACCT	ACTGAAGAATTGATGGCAGTGAT	TET2_15
Chr4	**TET2**	TGAGGCATCACTGCCATCAATT	GCACATGAGCTTTTGGTAAATGGTC	TET2_10
Chr4	**TET2**	TCTTCTTCACAGGTGCTTTCAA	AAGAGATGCCACCTTAGAGCAG	TET2_13
Chr4	**TET2**	GGATGTGGTCATAGAATAAAGTTATGCTCA	CAATTCTCAGGGTCAGATTTACAAATCAAA	TET2_1
Chr4	**TET2**	TTCGCAGAAGCAGCAGTGAAGA	CCTTTCAACCAAAGATTGGGCTTTC	TET2_6
Chr4	**TET2**	AGTCAATTGAAATAGTTCTGTGTGTGGTTA	GGGATCTTGCTTCTGGCAAACTTA	TET2_8
Chr4	**TET2**	ATAAAGGCACCATATATTGTGTTTGGGA	CAAGTAAGTTGTTACAATTGCTGCCAAT	TET2_5
Chr4	**TET2**	TGGTCTAAATACTAGTGAGTTTTCGGTGTA	GCAGTGTGAGAACAGACTCAACAG	TET2_7
Chr4	**TET2**	GACTTTGCTCTTATCTTTGCTTAATGGG	AGACTCTGTCTGAGGGTGATGT	TET2_3
Chr4	**TET2**	AGCTCTTAACCATGATAGAACTGCTTG	GGTGATTCCTATTGGGATTCTTTAAAGGG	TET2_2
Chr5	**NPM1**	GATGTCTATGAAGTGTTGTGGTTCCT	GACAGCCAGATATCAACTGTTACAGAA	NPM1_1
Chr7	**EZH2**	TGCAAATTCAGAATTTCAAACTGCATGT	CATTTTAATGCACCCACTATCTTCAGC	EZH2_1
Chr7	**EZH2**	CTGACTTGTTCACATAACAAACAACTATCC	AGAACTGTAACCAGTTGCATTTACAAAATC	EZH2_2
Chr8	**RAD21**	GAAACATTTCTAAGGATTTTCCTTGCACT	AGAACAATGTTCTACGCACATTTTGTTC	RAD21_1
Chr9	**JAK2**	GCAGGTCCATATAAAGGGACCAA	AGGCATTAGAAAGCCTGTAGTTTTACTTAC	JAK2_1
ChrX	**STAG2**	TTTTGTGAATTCATTGGCGTGTTAGTAC	GAGTGTCCTCTTAGTGGTCACTTTATAAAT	STAG2_1
ChrX	**PHF6**	ACAGTCCAGCTCACAACAACAT	AGAAATAAGTTTCAGTAACATCAGCATCCT	PHF6_1

Supplemental Table S1. *AML targeted sequencing panel.* List of the 25 genes and gene specific primer sequences for the 62 amplicon targeted sequencing panel. The panel was designed to include at least partial coverage for 18 of the 23 most significantly mutated genes in AML, including 14 of the top 15 most commonly mutated genes.
