# Supplementary material for: High-throughput single-cell DNA sequencing of acute myeloid leukemia tumors with droplet microfluidics
Source: Genome Res. 2018 Sep;28(9):1345–52. doi: 10.1101/gr.232272.117 (PMC6120635; doi:10.1101/gr.232272.117)
Supplement: Supplemental Material [file supp_gr.232272.117_Supplemental_Table_S2.pdf]

Gene	Transcript	Strand	Variant coordinates (gDNA/cDNA/protein)	Region
DNMT3A	NM_022552	-	Chr2:g.25457192G>A/c.2695C>T/p.R899C	cds_in_exon_23
DNMT3A	NM_022552	-	Chr2:g.25458546C>T/c.2597+30G>A/.	intron_between_exon_22_and_23
DNMT3A	NM_022552	-	Chr2:g.25463483G>A/c.2173+26C>T/.	intron_between_exon_18_and_19
SF3B1	NM_012433	-	Chr2:g.198266943C>T/c.2078-89G>A/.	intron_between_exon_14_and_15
IDH1	NM_001282387	-	Chr2:g.209113058delA/c.414+44delT/.	intron_between_exon_4_and_5
KIT	NM_000222	+	Chr4:g.55599436T>C/c.2484+78T>C/.	intron_between_exon_17_and_18
TET2	NM_001127208	+	Chr4:g.106196092C>T/c.4538-113C>T/.	intron_between_exon_10_and_11
NPM1	NM_002520	+	Chr5:g.170837526delT/c.847-5delT/.	intron_between_exon_10_and_11
EZH2	NM_001203247	-	Chr7:g.148504722delG/c.2241+21delC/.	3-UTR;noncoding_exon_20
EZH2	NM_001203247	-	Chr7:g.148504855_148504859dupGACTT/c.2181-61_2181-57dupAAGTC/.	intron_between_exon_19_and_20
FLT3	NM_004119	-	Chr13:g.28608255_28608275dupGATCATATTCATATTCTCTGA/c.1784_1804dupGAGAATATGAATATGATCTCA/p.R595_L601dupREYEYDL	cds_in_exon_14
TP53	NM_001126115	-	Chr17:g.7577180C>T/c.387-25G>A/.	intron_between_exon_3_and_4
TP53	NM_001126115	-	Chr17:g.7577407A>C/c.386+92T>G/.	intron_between_exon_3_and_4
TP53	NM_001126115	-	Chr17:g.7577427G>A/c.386+72C>T/.	intron_between_exon_3_and_4
TP53	NM_001126115	-	Chr17:g.7578115T>C/c.276+62A>G/.	intron_between_exon_2_and_3
TP53	NM_001126115	-	Chr17:g.7578394T>C/c.140A>G/p.H47R	cds_in_exon_1
ASXL1	NM_015338	+	Chr20:g.31022959T>C/c.2444T>C/p.L815P	cds_in_exon_12

Supplemental Table S2. *List of the sequence variants identified from the patient 1 AML diagnosis sample.* From this list, four non-synonymous coding region variants were identified in *ASXL1*, *TP53*, *DNMT3A* and *FLT3*.
