# Supplementary material for: High-throughput single-cell DNA sequencing of acute myeloid leukemia tumors with droplet microfluidics
Source: Genome Res. 2018 Sep;28(9):1345–52. doi: 10.1101/gr.232272.117 (PMC6120635; doi:10.1101/gr.232272.117)
Supplement: Supplemental Material [file supp_gr.232272.117_Supplemental_Table_S3.pdf]

Gene	Transcript	Strand	Variant coordinates (gDNA/cDNA/protein)	Region
NRAS	NM_002524	-	Chr1:g.115258745C>G/c.37G>C/p.G13R	cds_in_exon_2
SF3B1	NM_012433	-	Chr2:g.198266943C>T/c.2078-89G>A/.	intron_between_exon_14_and_15
SF3B1	XM_005246428	-	Chr2:g.198267246_198267247insG/c.1639+33_1639+34insC/.	intron_between_exon_15_and_16
IDH1	XM_005246524	-	Chr2:g.209113058delA/c.414+44delT/.	intron_between_exon_2_and_3
KIT	NM_000222	+	Chr4:g.55599436T>C/c.2484+78T>C/.	intron_between_exon_17_and_18
NPM1	NM_002520	+	Chr5:g.170837457A>G/c.847-74A>G/.	intron_between_exon_10_and_11
NPM1	NM_002520	+	Chr5:g.170837526delT/c.847-5delT/.	intron_between_exon_10_and_11
EZH2	NM_001203249	-	Chr7:g.148504718G>A/c.2088+20C>T/.	3-UTR;noncoding_exon_19
EZH2	NM_001203249	-	Chr7:g.148504855_148504859dupGACTT/c.2028-61_2028-57dupAAGTC/.	intron_between_exon_18_and_19
JAK2	NM_004972	+	Chr9:g.5073691delT/c.1777-7delT/.	intron_between_exon_13_and_14
WT1	XM_005253120	-	Chr11:g.32417945T>C/c.852A>G/p.R284R	cds_in_exon_6
WT1	XM_005253120	-	Chr11:g.32417962A>G/c.844-9T>C/.	intron_between_exon_5_and_6
FLT3	NM_004119	-	Chr13:g.28592546T>C/c.2541+58A>G/.	intron_between_exon_20_and_21
FLT3	NM_004119	-	Chr13:g.28602256C>T/c.2053+59G>A/.	intron_between_exon_16_and_17
IDH2	XM_005254893	-	Chr15:g.90631934C>T/c.263G>A/p.R140Q	cds_in_exon_4
TP53	NM_001126115	-	Chr17:g.7577180C>T/c.387-25G>A/.	intron_between_exon_3_and_4
TP53	NM_001126115	-	Chr17:g.7578115T>C/c.276+62A>G/.	intron_between_exon_2_and_3
SRSF2	NM_001195427	-	Chr17:g.74732959G>T/c.284C>A/p.P95H	cds_in_exon_1
ASXL1	NM_015338	+	Chr20:g.31022449dupG/c.1934dupG/p.G646Wfs*12	cds_in_exon_13
ASXL1	NM_015338	+	Chr20:g.31022959T>C/c.2444T>C/p.L815P	cds_in_exon_13

Supplemental Table S3. *List of the sequence variants identified from the patient 2 AML diagnosis sample.* From this list, five non-synonymous coding region variants were identified in *ASXL1*, *IDH2*, *NRAS* and *SRSF2*. These variants were identified with a targeted single-cell sequencing panel that was slightly modified from the panel shown in Table S1 with amplicons covering *CBL*, *MIR142*, *TET2*, *PHF6*, *RAD21* and *STAG2* removed. The *SRSF2* mutation was not considered for further analysis due to the majority of cells identified not having any sequence coverage at this position.
