# Supplementary material for: High-throughput single-cell DNA sequencing of acute myeloid leukemia tumors with droplet microfluidics
Source: Genome Res. 2018 Sep;28(9):1345–52. doi: 10.1101/gr.232272.117 (PMC6120635; doi:10.1101/gr.232272.117)
Supplement: Supplemental Material [file supp_gr.232272.117_Supplemental_Table_S4.pdf]

Gene name									
ABCC9	CALR	CUL5	FANCD2	HIST1H2BF	LEF1	NBN	PLA2G2D	SF3B1	TINF2 (TIN2)
ABL1	CARD11	CUX1	FANCE	HIST1H3D	LRP1B	NCOR1	PLCG2	SFRS1	TLR2
ACTG1	CBL	CYLD	FANCG	HIST1H4D	LTB	NCOR2	POT1	SFRS7	TLR9
AKT1	CBLB	DAXX	FANCI	HNRNPK	LUC7L2	NF1	POU2AF1	SGK1	TNFAIP3
ANKRD11	CCND1	DCLRE1C	FANCL	HRAS	LYN	NFE2	PRDM1	SH2B3	TNFRSF14
ARID1A	CCND3	DDX3X	FAS	ICOS	MALT1	NFKB1	PRKCB	SHH	TNKS
ARID1B	CD200	DIS3	FAT1	ID3	MAP2K1	NFKB2	PTEN	SMAD2	TOX
ARID2	CD274	DKC1	FAT3	IDH1	MAPK1	NFKBIA	PTPN1	SMC1A	TP53
ARID5B	CD58	DLC1	FBXW7	IDH2	MAX	NFKBIE	PTPN11	SMC3	TRAF3
ARPP21	CD79A	DNM2	FGFR3	IKBKA	MDM2	NOTCH1	RAD21	SMC5	TRAF6
ASXL1	CD79B	DNMT1	FLI1	IKZF1	MED12	NOTCH2	RAD51C	SNX7	TYK2
ATF7IP	CDK4	DNMT3A	FLT3	IKZF2	MEF2B	NPM1	RAG1	SOCS1	TYK3
ATM	CDKN2A	DNMT3B	FNDC3A	IKZF3	MEF2C	NR3C2	RAG2	SOX5	U2AF1
ATRX	CDKN2B	EBF1	FOXP1	IL7R	MGA	NRAS	RASA2	SP140	U2AF2
B2M	CDKN2C	ECT2L	FYN	IRAK1	miR125a	NSD2	RB1	SPEN	UBR5
BCL10	CEBPA	EED	G6PC3	IRAK4	miR-142	NT5C2	REL	SPIB	USP29
BCL2	CEBPE	EGR1	GAB2	IRF1	mIR155	PAG1	RELA	SRSF2	VPREB1
BCL6	CHD2	EGR2	GATA1	IRF4	mIR15a	PALB2	RELB	STAG1	WHSC1
BCL7A	CHK2	ELANE	GATA2	IRF7	mIR16-1	PAX5	RELN	STAG2	WHSC1L1
BCOR	CIITA	EP300	GATA3	ITPKB	MIR17HG	PDCD1	RHOA	STAT1	WT1
BCR	CNOT3	EPHA7	GCET2	JAK1	mIR21	PDCD1LG2	RIPK1	STAT3	XPO1
BIRC3	CREBBP	EPOR	GFI1B	JAK2	mir34b	PDGFRB	ROBO1	SUZ12	ZAP70
BLK	CRLF2	ERG	GNA13	JAK3	mir34c	PEG3	ROR1	SYK	ZMYM2
BMI1	CSF2RA	ETV6	GNAS	JARID2	MLL	PHF6	RPL10	TBL1XR1	ZMYM3
BRAF	CSF3R	EZH2	GNB1	KDM4C	MLL2	PHIP	RPL5	TCF3	ZRSR2
BRIP1	CTBP1	FAM46C	GPRC5A	KDM6A	MLL3	PIGA	RUNX1	TERC	
BTG1	CTBP2	FAM5C	HAX1	KIT	MPL	PIK3CA	RUNX2	TERT	
BTK	CTCF	FANCA	HIST1H1E	KLHL6	MS4A1	PIK3CB	SAMHD1	TET1	
BTLA	CTLA4	FANCB	HIST1H2AD	KRAS	MYB	PIK3CG	SETBP1	TET2	
C22orf194	CTNNA1	FANCC	HIST1H2BE	LAMB4	MYD88	PIK3R1	SETD2	TGDS	

Supplemental Table S4. *List of the 295 genes that were targeted for bulk sequencing.*
